# Triglyceride-glucose index, renal function, sleep duration, and myocardial infarction: a UK biobank cohort study

**DOI:** 10.3389/fnut.2025.1646627

**Published:** 2025-08-29

**Authors:** Zeyan Liu, Jinbo Wang, Xiaodong Pan, Pan Zhang, Min Yang, Ye Zhang

**Affiliations:** ^1^Department of Emergency Internal Medicine, Second Affiliated Hospital of Anhui Medical University, Anhui, Hefei, China; ^2^Chest Pain Center, Second Affiliated Hospital of Anhui Medical University, Anhui, Hefei, China; ^3^Division of Life Sciences and Medicine, Department of Neurology, The First Affiliated Hospital of USTC, University of Science and Technology of China, Hefei, Anhui, China; ^4^Department of Intensive Care Unit II, Second Affiliated Hospital of Anhui Medical University, Anhui, Hefei, China; ^5^Department of Anesthesiology and Perioperative Medicine, Second Affiliated Hospital of Anhui Medical University, Anhui, Hefei, China

**Keywords:** triglyceride-glucose index, myocardial infarction, renal function, sleep duration, insulin resistance

## Abstract

**Background:**

Myocardial infarction (MI) remains a leading cause of cardiovascular morbidity and mortality globally. Insulin resistance, renal function, and sleep duration are key risk factors, yet their combined impact on MI risk is underexplored.

**Methods:**

In this UK Biobank cohort study, 415,757 participants were included. Cox proportional hazards models estimated hazard ratios (HRs) for MI risk across quartiles of triglyceride-glucose (TyG) indices (TyG, TyG-BMI, TyG-WC, TyG-WHTR), stratified by eGFR and sleep duration categories. Mediation analyses evaluated interrelationships among TyG indices, eGFR, sleep duration, and MI.

**Results:**

Over follow-up, 13,484 participants developed MI. Higher TyG indices were associated with increased MI risk; TyG-WHTR showed the strongest effect (Q4 vs. Q1, HR: 1.90, 95% CI: 1.76–2.06). Reduced eGFR (<60) was linked to higher MI risk (HR: 1.71, 95% CI: 1.51–1.93), as were short (<7 h; HR: 1.20, 95% CI: 1.11–1.29) and long (>9 h; HR: 1.43, 95% CI: 1.22–1.68) sleep durations. TyG-MI associations were significant in participants with preserved renal function (eGFR ≥60) and short/normal sleep. Mediation analyses revealed that eGFR and sleep duration partially mediated the associations between TyG indices and MI risk, and vice versa, indicating complex interrelations.

**Conclusion:**

TyG-related indices are valuable predictors of MI risk, particularly in individuals with preserved renal function and typical sleep duration. The interplay among insulin resistance, renal function, and sleep patterns underscores the importance of integrated metabolic and lifestyle factors in cardiovascular risk stratification.

## Introduction

Myocardial infarction (MI) remains a major public health challenge, accounting for a substantial proportion of cardiovascular-related morbidity and mortality globally (1). The rising prevalence of metabolic disorders, particularly insulin resistance, has underscored its pivotal role in the pathogenesis of MI (2).

Insulin resistance, a key metabolic dysfunction, is heavily influenced by dietary factors—particularly high intake of refined carbohydrates, saturated fats, and excessive caloric consumption (3, 4). These nutritional exposures drive elevations in fasting glucose and triglyceride levels, which are captured by the triglyceride-glucose (TyG) index (5). Serving as a nutrition-sensitive proxy for insulin resistance, various TyG-derived indices like TyG-BMI, TyG-WC, and TyG-WHTR have been devised. These indices amalgamate anthropometric measurements to encapsulate the intertwined impacts of insulin resistance and obesity-related risk factors (6, 7).

In addition to conventional metabolic indices, sleep duration has emerged as a critical determinant of glucose metabolism and lipid homeostasis (8). Both short (<7 h) and long (>9 h) sleep durations have been independently associated with elevated cardiovascular risk, likely through mechanisms involving increased sympathetic activity, impaired glucose regulation, and systemic inflammation (9, 10). Such deviations in sleep patterns may not only signal underlying nutritional imbalances and metabolic stress but may also contribute to their progression, highlighting sleep as a modifiable factor in public health nutrition.

Similarly, accumulating evidence underscores the pivotal role of renal function in modulating cardiovascular risk. Chronic kidney disease (CKD), commonly assessed via estimated glomerular filtration rate (eGFR), is a well-established risk factor for myocardial infarction (MI), as it contributes to metabolic disturbances, oxidative stress, and vascular calcification (11).

Despite the interrelated nature of these factors, the joint effects of TyG indices, renal function, and sleep duration on MI risk have not been adequately investigated. In this study, we examine the associations between TyG-related indices—including TyG, TyG-BMI, TyG-WC, and TyG-WHTR and the risk of incident MI. We specifically assess how these relationships may vary across levels of renal function and sleep duration, both of which are modifiable through behavioral and nutritional interventions. We further explore potential mediating roles of eGFR, sleep duration, and TyG-related indices in shaping MI risk. Understanding these interrelationships may offer new insights into nutrition-sensitive pathways contributing to cardiovascular disease and help identify at-risk individuals who could benefit from targeted preventive strategies.

## Methods

### Study population

This research utilized data from the UK Biobank, a prospective cohort study that recruited over 500,000 adults aged 40–69 years across the United Kingdom between 2006 and 2010 (12). Participants were enrolled from 22 assessment centers in England, Scotland, and Wales, where they completed detailed questionnaires, underwent physical examinations, and provided biological samples, including blood for biomarker analysis. The UK Biobank was designed to explore the contributions of genetic, lifestyle, and environmental factors to the development of complex diseases. Ethical approval was obtained from the North West Multi-Centre Research Ethics Committee (reference: 21/NW/0157), and all participants provided written informed consent. For this analysis, we excluded individuals with missing covariates (*n* = 75,087), pre-existing cardiovascular disease at baseline (*n* = 8,379), and those lacking sleep duration and renal function data (*n* = 3,046), resulting in a final study population of 415,757 participants.

### Measurement of TyG and related indices

Four insulin resistance indices were evaluated: the TyG index, TyG-BMI, TyG-WC, and TyG-WHTR. These indices were derived using fasting blood measurements of triglycerides and glucose, along with anthropometric data collected at baseline. The TyG index was calculated as:

TyG = Ln[Triglycerides (mg/dL) × Fasting Glucose (mg/dL)/2].

The derived indices were computed as follows: TyG-BMI = TyG × Body Mass Index (BMI, kg/m^2^), TyG-WC = TyG × Waist Circumference (WC, cm), TyG-WHTR = TyG × Waist-to-Height Ratio (WHTR, calculated as WC/height).

Triglyceride and glucose levels were measured using standardized laboratory protocols, and anthropometric measurements were obtained by trained staff during the baseline visit.

### Evaluation of renal function

Renal function was assessed using the estimated glomerular filtration rate (eGFR), calculated with the Chronic Kidney Disease Epidemiology Collaboration (CKD-EPI) equation (13):

eGFR = 142 × min(Scr/*κ*, 1)^*α* × max(Scr/κ, 1)^(−1.200) × 0.9938^(age) × 1.012 (if female),

where Scr is serum creatinine (mg/dL), κ is 0.7 for females and 0.9 for males, and α is −0.241 for females and −0.302 for males. Serum creatinine was measured using enzymatic methods. Participants were categorized into three eGFR groups: ≥90, 60–90, and <60 mL/min/1.73 m^2^, reflecting normal, mildly reduced, and significantly reduced renal function, respectively.

### Assessment of sleep duration

Sleep duration was self-reported by participants via a touchscreen questionnaire at baseline, where they were asked to report their average sleep duration per 24 h, including naps. Responses were categorized into three groups based on established guidelines: short sleep (<7 h), normal sleep (7–9 h), and long sleep (>9 h) (14, 15).

### Ascertainment of myocardial infarction

The primary outcome was incident MI, identified using International Classification of Diseases, 10th Revision (ICD-10) code I21. MI events were ascertained through linkage to hospital inpatient records, primary care data, and national death registries. Follow-up time was calculated from the baseline assessment date until the first occurrence of MI, death, loss to follow-up, or the end of the study period, whichever occurred first.

### Covariate assessment

A comprehensive set of covariates was collected at baseline to adjust for potential confounders. These included demographic factors (age, sex, ethnicity), socioeconomic status [Townsend Deprivation Index, derived from postal code data, with higher values indicating greater deprivation (16)], lifestyle factors [smoking status (never, former, current), alcohol consumption (never, former, current)], and physical activity [measured in metabolic equivalent of task (MET) hours per week]. Clinical variables included systolic and diastolic blood pressure (measured using automated devices), history of hypertension (defined as self-reported diagnosis, use of antihypertensive medication, or blood pressure ≥140/90 mmHg), history of stroke, high cholesterol, and glucose metabolic status. Glucose metabolism was categorized per American Diabetes Association criteria (17): normal glucose regulation [fasting blood glucose (FBG) < 5.6 mmol/L and HbA1c < 5.7%, no glucose-lowering medication], prediabetes (FBG 5.6–6.9 mmol/L or HbA1c 5.7–6.4%, no medication), and diabetes (FBG ≥ 7.0 mmol/L, HbA1c ≥ 6.5%, or use of glucose-lowering medication).

### Statistical analysis

Baseline characteristics were summarized using descriptive statistics: means ± standard deviations for normally distributed continuous variables, medians (interquartile ranges) for skewed continuous variables, and frequencies (percentages) for categorical variables.

Cox proportional hazards regression models were used to estimate hazard ratios (HRs) and 95% confidence intervals (CIs) for the association between TyG-related indices, renal function, sleep duration and MI risk. The proportional hazards assumption was tested using Schoenfeld residuals, and no significant violations were detected. Two models were constructed: Model 1 adjusted for age, sex, ethnicity, Townsend Deprivation Index; Model 2 further adjusted for smoking status, alcohol consumption, history of hypertension, high cholesterol, glucose metabolic status, physical activity, systolic and diastolic blood pressure, and history of stroke. TyG-related indices were analyzed both as continuous variables (per unit increase) and as categorical variables (quartiles, with Q1 as the reference). Kaplan–Meier survival curves were generated to visualize the cumulative incidence of MI across TyG quartiles. Nonlinear associations were explored using restricted cubic spline analysis with four knots, adjusted for Model 2 covariates, to assess dose–response relationships. Subgroup analyses were conducted to evaluate the associations of TyG-related indices with MI risk across eGFR categories (≥90, 60–90, <60 mL/min/1.73 m^2^) and sleep duration categories (short, normal, long). We performed additional exploratory analyses, stratified by sex (female, male) and ethnicity (White, Black, Asian, Other), to investigate whether the relationships between TyG-related indices and incident MI vary across these groups.

To further evaluate the predictive ability of TyG-related indices, we conducted receiver operating characteristic (ROC) curve analyses, calculating the area under the curve (AUC) with 95% CIs. The optimal cut-off points for each index were determined using the Youden index, and corresponding sensitivity and specificity values were reported.

Sensitivity analyses were performed to test the robustness of findings by (1) excluding MI events within the first 2 years of follow-up to minimize reverse causation, and (2) additionally adjusting for the use of antidiabetic and lipid-lowering medications in Model 2.

Additionally, we performed a mediation analysis to investigate whether eGFR, sleep duration, or TyG-related indices mediate the associations between each other and MI risk. Traditional mediation analysis relies on parametric regression models and evaluates three distinct effects: (I) the total effect, which represents the overall impact of the independent variable (X) on the outcome (Y), encompassing both direct and indirect pathways; (II) the direct effect, which captures the effect of X on Y through pathways independent of the mediator; and (III) the indirect effect, which reflects the influence of X on Y via the mediator. When the total, direct, and indirect effects are directionally consistent, a mediation ratio can be computed to estimate the proportion of the total effect explained by the mediator. Given the nonlinear (U-shaped) relationship observed between sleep duration and MI risk, mediation analyses involving sleep duration were performed separately for participants with ≤7.5 h and >7.5 h of sleep per night to approximate linearity and meet mediation model assumptions.

All analyses were conducted using R (version 4.2.3), and a two-sided *p*-value <0.05 was considered statistically significant.

## Results

### Baseline characteristics

After applying exclusion criteria—75,087 participants with missing key covariates, 8,379 with prior cardiovascular disease at baseline, and 3,046 lacking sleep duration and renal function data—a total of 415,757 participants were included in the analysis. During follow-up, 13,484 (3.2%) participants developed incident MI. The participant selection process is illustrated in Supplementary Figure S1. The overall cohort was predominantly middle-aged, with a balanced gender distribution. Participants who developed MI were generally older and more likely to be male compared to those who did not develop MI. All TyG-related indices, including TyG, TyG-BMI, TyG-WC, and TyG-WHTR, were significantly higher in the MI group than in the non-MI group (all *p* < 0.001). Detailed demographic characteristics and TyG-related index values are presented in Table 1.

**Table 1:** Baseline characteristics of participants.

	Overall	Non-myocardial infarction	Myocardial infarction	*P*
Sample size	415,757	402,273	13,484	
Age, years [median (IQR)]	58.00 [50.00, 63.00]	57.00 [50.00, 63.00]	61.00 [55.00, 65.00]	<0.001
Sex, n (%)				<0.001
Female	226,507 (54.5)	222,501 (55.3)	4006 (29.7)	
Male	189,250 (45.5)	179,772 (44.7)	9478 (70.3)	
Ethnicity, n (%)				<0.001
White	396,436 (95.4)	383,599 (95.4)	12,837 (95.2)	
Black	9752 (2.3)	9344 (2.3)	408 (3.0)	
Asian	2783 (0.7)	2744 (0.7)	39 (0.3)	
Other	6782 (1.6)	6582 (1.6)	200 (1.5)	
TDI [median (IQR)]	−2.17 [−3.67, 0.46]	−2.18 [−3.67, 0.44]	−1.93 [−3.52, 0.97]	<0.001
Smoking, n (%)				<0.001
Never	228,410 (55.1)	222,747 (55.6)	5663 (42.2)	
Former	142,522 (34.4)	137,104 (34.2)	5418 (40.4)	
Current	43,346 (10.5)	41,010 (10.2)	2336 (17.4)	
Alcohol status, n (%)				<0.001
Never	17,964 (4.3)	17,298 (4.3)	666 (4.9)	
Former	14,500 (3.5)	13,775 (3.4)	725 (5.4)	
Current	382,852 (92.2)	370,771 (92.3)	12,081 (89.7)	
MET h/week, median [IQR]	29.90 [13.65, 59.55]	29.90 [13.70, 59.53]	29.55 [12.32, 60.98]	0.024
Hypertension history, n (%)	107,709 (25.9)	102,059 (25.4)	5650 (41.9)	<0.001
High_cholesterol history, n(%)	47,697 (11.5)	45,012 (11.2)	2685 (19.9)	<0.001
Stroke history, n(%)	4864 (1.2)	4498 (1.1)	366 (2.7)	<0.001
GMS, n (%)				<0.001
NGR	335,907 (84.5)	325,818 (84.8)	10,089 (77.6)	
Pre-DM	46,562 (11.7)	44,702 (11.6)	1860 (14.3)	
DM	14,965 (3.8)	13,920 (3.6)	1045 (8.0)	
Sleep time, n (%)				<0.001
Short	102,325 (24.6)	98,657 (24.5)	3668 (27.2)	
Normal	306,094 (73.6)	296,674 (73.7)	9420 (69.9)	
Long	7338 (1.8)	6942 (1.7)	396 (2.9)	
BMI [median (IQR)]	26.70 [24.12, 29.84]	26.66 [24.08, 29.79]	27.91 [25.34, 31.04]	<0.001
WC [median (IQR)]	90.00 [80.00, 99.00]	89.00 [80.00, 98.00]	96.00 [88.00, 104.00]	<0.001
TyG [median (IQR)]	8.68 [8.31, 9.07]	8.67 [8.30, 9.06]	8.89 [8.51, 9.28]	<0.001
TyG-BMI [median (IQR)]	232.90 [204.36, 266.82]	232.35 [203.88, 266.18]	249.08 [220.80, 282.06]	<0.001
TyG-WC [median (IQR)]	780.06 [679.43, 883.03]	777.53 [677.25, 880.36]	851.11 [761.16, 945.34]	<0.001
TyG-WHTR [median (IQR)]	4.62 [4.07, 5.19]	4.61 [4.06, 5.18]	4.97 [4.48, 5.53]	<0.001
Glucose, mg/dL [median (IQR)]	88.77 [82.86, 95.63]	88.73 [82.84, 95.56]	90.05 [83.43, 98.57]	<0.001
TG, mg/dL [median (IQR)]	130.91 [92.29, 189.54]	130.11 [91.85, 188.30]	158.27 [109.74, 224.17]	<0.001
Renal function				<0.001
eGFR<60	5921 (1.4)	5463 (1.4)	458 (3.4)	
eGFR 60–90	119,156 (28.7)	114,457 (28.5)	4699 (34.9)	
eGFR>90	290,631 (69.9)	282,306 (70.2)	8325 (61.7)	

TDI, Townsend Deprivation Index; BMI, body mass index; WC, waist circumference; TG, triglycerides; TyG, triglyceride-glucose index; WHTR, Waist-to-Height Ratio; GMS, glucose metabolic states; NGR, normal glucose regulation; Pre-DM, prediabetes mellitus; DM, diabetes mellitus.

### Association between TyG-related indices and MI risk

Of the 415,757 participants, 13,484 (3.2%) experienced a first incident MI during follow-up. Kaplan–Meier survival analysis demonstrated a graded increase in MI incidence across quartiles (Q1–Q4) for TyG, TyG-BMI, TyG-WC, and TyG-WHTR indices, with statistically significant differences (log-rank *p* < 0.001, Supplementary Figure S2). The HRs and 95%CIs for the associations between TyG-related indices and MI risk, assessed using two Cox regression models, are shown in Table 2.

**Table 2:** Association of TyG-related indices with myocardial infarction.

Categories	Model 1		Model 2	
	HR (95% CI)	*p*-value	HR (95% CI)	*P*-value
TyG				
Continuous variable per unit	1.51(1.47–1.56)	<0.001	1.33(1.27–1.38)	<0.001
Quartile				
Q1	Reference		Reference	
Q2	1.18(1.11–1.25)	<0.001	1.14(1.06–1.22)	<0.001
Q3	1.39(1.32–1.47)	<0.001	1.27(1.19–1.36)	<0.001
Q4	1.8(1.71–1.9)	<0.001	1.5(1.4–1.6)	<0.001
TyG-BMI				
Continuous variable per unit	1(1–1)	<0.001	1(1–1)	<0.001
Quartile				
Q1	Reference		Reference	
Q2	1.29(1.22–1.37)	<0.001	1.28(1.19–1.37)	<0.001
Q3	1.49(1.41–1.58)	<0.001	1.4(1.31–1.51)	<0.001
Q4	1.99(1.88–2.1)	<0.001	1.68(1.56–1.8)	<0.001
TyG-WC				
Continuous variable per unit	1(1–1)	<0.001	1(1–1)	<0.001
Quartile				
Q1	Reference		Reference	
Q2	1.4(1.31–1.5)	<0.001	1.37(1.26–1.49)	<0.001
Q3	1.72(1.61–1.84)	<0.001	1.6(1.48–1.74)	<0.001
Q4	2.2(2.06–2.35)	<0.001	1.82(1.67–1.98)	<0.001
TyG-WHTR				
Continuous variable per unit	1.42(1.39–1.45)	<0.001	1.29(1.25–1.33)	<0.001
Quartile				
Q1	Reference		Reference	
Q2	1.44(1.35–1.54)	<0.001	1.4(1.29–1.51)	<0.001
Q3	1.78(1.67–1.89)	<0.001	1.64(1.52–1.77)	<0.001
Q4	2.31(2.18–2.46)	<0.001	1.9(1.76–2.06)	<0.001

Model 1 adjusted for age, sex, ethnicity, Townsend Deprivation Index. Model 2 further adjusted for smoking status, alcohol consumption, history of hypertension and high_cholesterol, SBP, DBP, history of stroke, and diabetes status. TyG, triglyceride-glucose index; BMI, body mass index; WC, waist circumference; WHTR, Waist-to-Height Ratio. HR, hazard ratios; CI, confidence intervals.

For the TyG index, each unit increase as a continuous variable was associated with an elevated MI risk in both models. In quartile analyses, participants in the highest quartile (Q4) had a significantly higher MI risk compared to Q1. After further adjusting for BMI, TyG remains associated with an increased risk of MI (Supplementary Table S1). Similarly, TyG-BMI, TyG-WC, and TyG-WHTR showed significant increases in MI risk with higher values, with the strongest associations observed in Q4 compared to Q1, and significant trends in intermediate quartiles (all *p* < 0.001).

**Figure 1:**
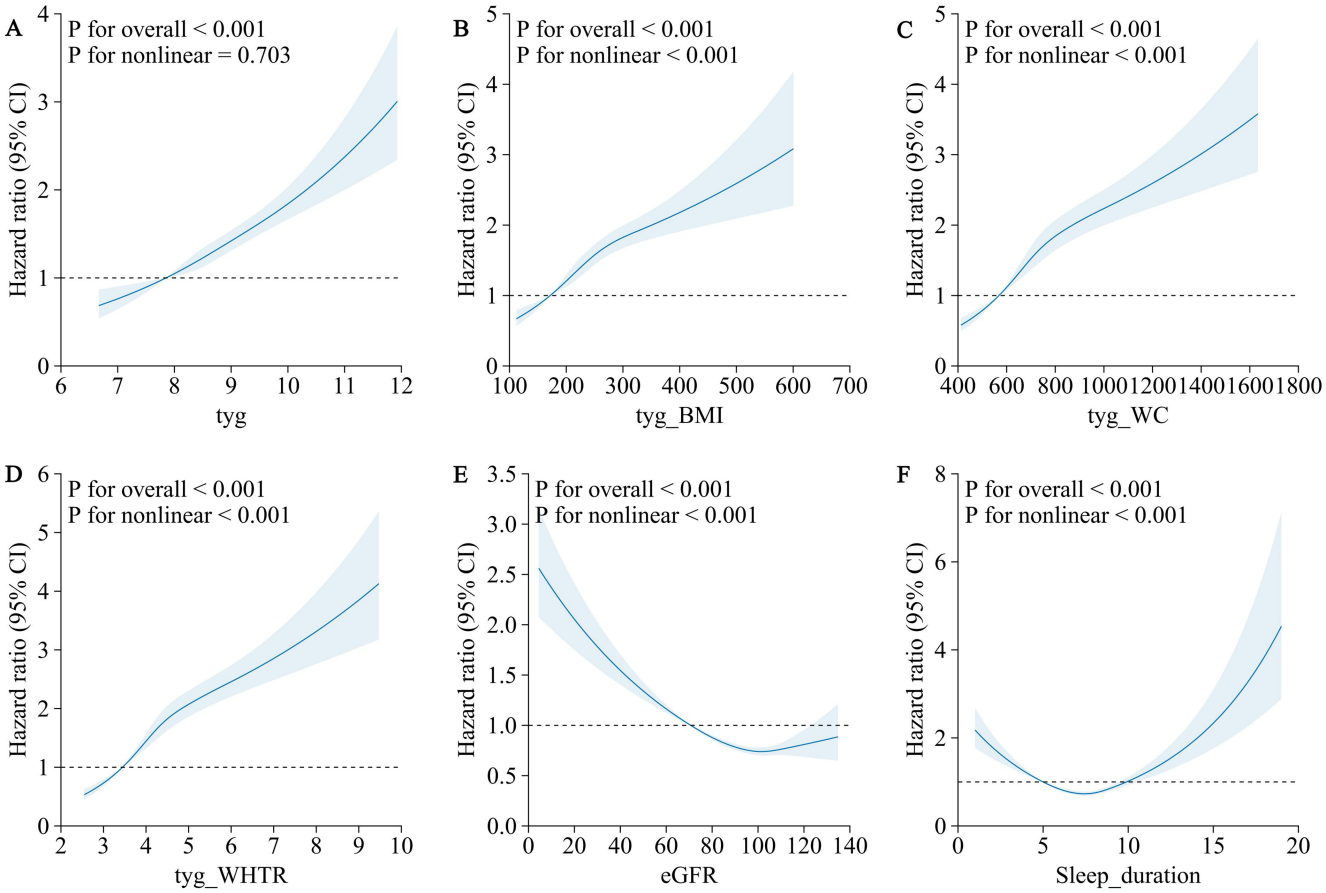
Association of TyG-related indexes (TyG, TyG-BMI, TyG-WC, and TyG-WHTR), renal function, sleep duration and the risk of myocardial infarction using a multivariable-adjusted restricted cubic spines model. **(A)** TyG **(B)** TyG-BMI **(C)** TyG-WC **(D)** TyG-WHTR **(E)** eGFR **(F)** sleep duration.

Restricted cubic spline analyses confirmed significant dose–response relationships for all TyG-related indices (P for overall association < 0.001). Nonlinear associations were observed for TyG-BMI, TyG-WC, and TyG-WHTR (P for nonlinearity < 0.001 for each), while the TyG index exhibited a linear relationship with MI risk (P for nonlinearity = 0.703, Figure 1).

### Predictive ability of TyG-related indices for MI

To further explore the predictive ability of TyG-related indices for MI, we performed ROC curve analyses and determined the optimal cut-off values based on the Youden index (Supplementary Table S2). The AUC values ranged from 0.601 to 0.642, with TyG-WC showing the highest discriminative ability (AUC: 0.642, 95% CI: 0.637–0.646). Optimal cut-off points were 8.70 for TyG, 225.51 for TyG-BMI, 777.98 for TyG-WC, and 4.61 for TyG-WHTR. Sensitivity ranged from 0.628 to 0.712, while specificity ranged from 0.440 to 0.523.

### Associations of renal function and sleep duration with MI risk

The associations of renal function and sleep duration with MI risk are presented in Table 3. Compared to participants with eGFR >90 mL/min/1.73 m^2^, those with eGFR <60 mL/min/1.73 m^2^ had a significantly higher MI risk, while those with eGFR 60–90 mL/min/1.73 m^2^ showed a modest increase. For sleep duration, relative to normal sleep (7–9 h), both short sleep (<7 h), and long sleep (>9 h) were associated with increased MI risk, with a stronger association observed for long sleep.

**Table 3:** Association of renal function, sleep duration with myocardial infarction.

	Model 1		Model 2	
	HR (95%CI)	*P*	HR (95%CI)	*P*
Renal function				
eGFR>90	Ref		Ref	
eGFR<60	2.05(1.86–2.25)	<0.001	1.71(1.51–1.93)	<0.001
eGFR 60–90	1.14(1.1–1.18)	<0.001	1.18(1.13–1.23)	<0.001
Sleep duration				
Normal	Ref		Ref	
Short	1.18(1.14–1.23)	<0.001	1.2(1.11–1.29)	<0.001
Long	1.66(1.5–1.84)	<0.001	1.43(1.22–1.68)	<0.001

Model 1 adjusted for age, sex, ethnicity, Townsend Deprivation Index. Model 2 further adjusted for smoking status, alcohol consumption, history of hypertension and high_cholesterol, SBP, DBP, history of stroke, and diabetes status. HR, hazard ratios; CI, confidence intervals.

Restricted cubic spline analyses revealed nonlinear dose–response relationships for both renal function and sleep duration with MI risk (P for nonlinearity < 0.001 for both). The association between eGFR and MI risk followed an L-shaped pattern, with a sharp increase in risk as eGFR fell below 90 mL/min/1.73 m^2^ (Figure 1). The relationship between sleep duration and MI risk exhibited a U-shaped pattern, with elevated risks at both short and long sleep durations (Figure 1).

### Stratified analysis by renal function

**Figure 2:**
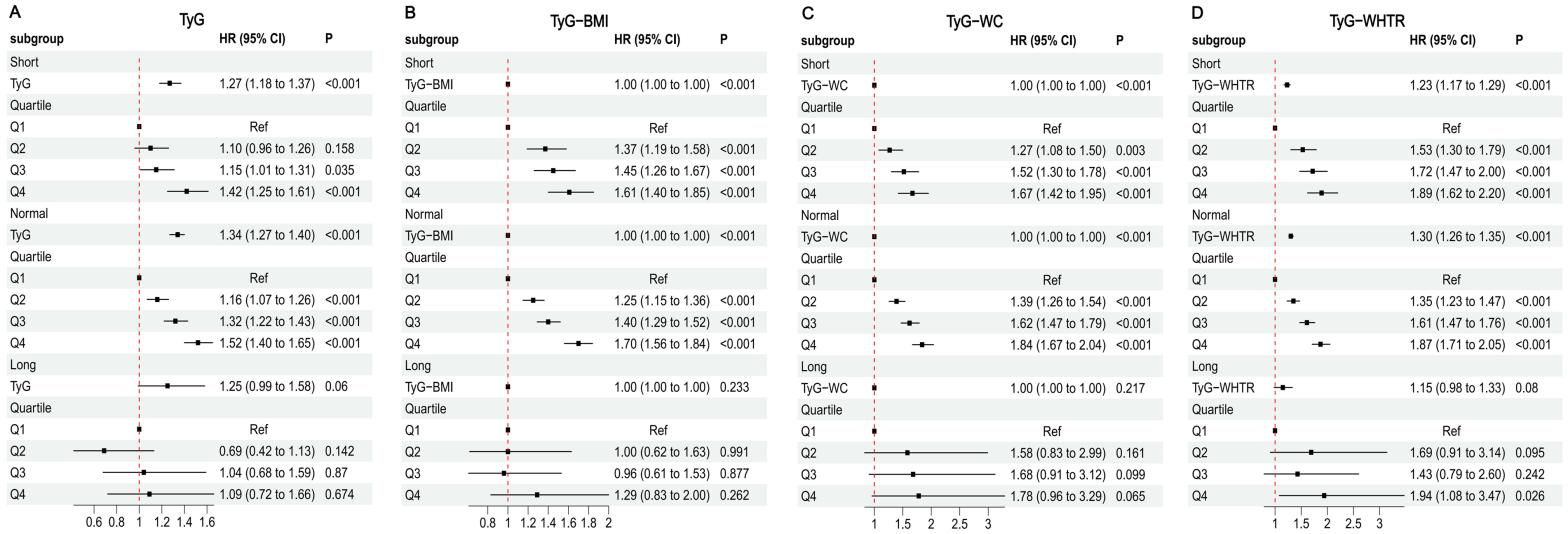
Associations between TyG-related indices and MI in different kidney function states. **(A)** TyG **(B)** TyG-BMI **(C)** TyG-WC **(D)** TyG-WHTR.

Stratified analyses by renal function status are shown in Figure 2. TyG-related indices were significantly associated with MI risk in participants with preserved renal function (eGFR ≥60 mL/min/1.73 m^2^). In both the eGFR 60–90 and eGFR ≥90 subgroups, higher quartiles of TyG, TyG-BMI, TyG-WC, and TyG-WHTR were associated with increased MI risk, with the strongest effects in Q4. In contrast, no significant associations were observed among participants with severely reduced renal function (eGFR <60 mL/min/1.73 m^2^, all *p* > 0.05). Kaplan–Meier curves further corroborated these findings (Supplementary Figure S2).

Restricted cubic spline analyses confirmed a significant dose–response relationship between TyG-related indices and MI risk in participants with eGFR ≥60 mL/min/1.73 m^2^ (P for overall association < 0.001), but not in those with eGFR <60 mL/min/1.73 m^2^ (P for overall association > 0.05) (Supplementary Figure S3).

### Stratified analysis by sleep duration

**Figure 3:**
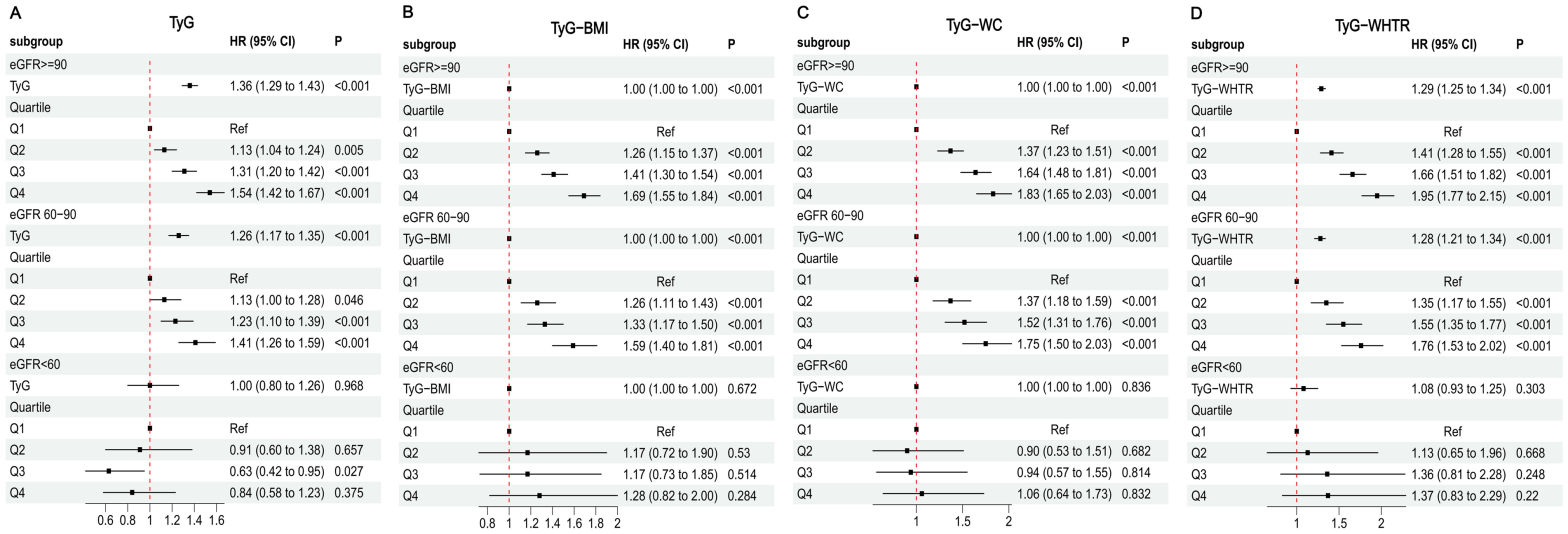
Associations between TyG-related indices and MI in different sleep duration. **(A)** TyG **(B)** TyG-BMI **(C)** TyG-WC **(D)** TyG-WHTR.

The associations between TyG-related indices and MI risk, stratified by sleep duration, are presented in Figure 3. In the short sleep (<7 h) and normal sleep (7–9 h) subgroups, higher values of TyG, TyG-BMI, TyG-WC, and TyG-WHTR were associated with increased MI risk, with significant effects observed in Q4 compared to Q1. The normal sleep subgroup generally showed stronger associations. In the long sleep (>9 h) subgroup, no significant associations were observed for any TyG-related indices (all *p* > 0.05). These findings were supported by Kaplan–Meier curves (Supplementary Figure S4).

Restricted cubic spline analyses indicated a significant dose–response relationship between TyG-related indices and MI risk in the short and normal sleep subgroups (P for overall association < 0.001), but not in the long sleep subgroup (P for overall association > 0.05) (Supplementary Figure S5).

### Exploratory analyses

We further conducted additional exploratory subgroup analyses stratified by sex and ethnicity to examine whether the associations between TyG-related indices and incident MI differ across these groups.

The associations between TyG-related indices and MI risk were observed in both females and males, with hazard ratios (HRs) for the highest vs. lowest quartile (Q4 vs. Q1) ranging from 1.53–1.81 in females and 1.37–1.85 in males. Although effect sizes were slightly higher in females for the TyG index itself, formal interaction testing did not reveal statistically significant sex–TyG interactions, suggesting similar patterns across sexes (Supplementary Table S3).

In White participants, all TyG-related indices showed significant positive associations with MI risk. In Black participants, the magnitude of association was generally higher (Supplementary Table S4).

### Sensitivity analysis

Sensitivity analysis was conducted to validate the robustness of our findings. After excluding participants who developed MI within the first 2 years of follow-up, or further adjusting for the use of antidiabetic and lipid-lowering medications, Cox regression analyses reaffirmed the primary results, highlighting the consistency of the observed associations (Supplementary Tables S5–S8).

### Mediation analysis of renal function, sleep duration, and TyG-related indices in MI risk

Mediation analyses were conducted to explore the mediating effect of eGFR and sleep duration on the associations of TyG-related indices with MI risk (Supplementary Table S9) and the mediating effect of insulin resistance indices on the associations of renal function and sleep duration with MI (Supplementary Table S10).

Given the U-shaped association between sleep duration and MI risk, mediation analyses for sleep duration were conducted separately for participants with ≤7.5 h and >7.5 h of sleep to better meet the linearity assumption. In these subgroup analyses, eGFR mediated 2.05–5.33% of the association between TyG-related indices and MI, whereas sleep duration mediated 2.12–4.79% in the ≤7.5 h group and 3.75–4.79% in the >7.5 h group. Conversely, TyG-related indices mediated 3.11–9.78% of the association between eGFR and MI, 3.97–12.50% for sleep duration ≤7.5 h, and 6.04–12.82% for sleep duration >7.5 h.

## Discussion

This study comprehensively evaluated the associations between TyG-related indices (TyG, TyG-BMI, TyG-WC, and TyG-WHTR) and incident MI in a large UK Biobank cohort, with particular attention to the modifying and mediating roles of renal function and sleep duration. Higher TyG-related indices were significantly associated with increased MI risk, showing dose–response patterns in most analyses. The associations were stronger in participants with preserved renal function and in those with short or normal sleep durations, and attenuated in individuals with impaired renal function or prolonged sleep. These results highlight the complex interplay between metabolic markers, renal function, and sleep patterns in MI pathogenesis, offering important implications for risk stratification and preventive strategies.

The TyG index and its derived indices (TyG-BMI, TyG-WC, and TyG-WHTR) were consistently associated with an elevated MI risk, aligning with previous studies that have established the TyG index as a reliable surrogate marker of insulin resistance and a predictor of cardiovascular events (18, 19). The TyG index reflects the combined effects of dyslipidemia and hyperglycemia, both of which are central to the development of atherosclerosis through mechanisms such as endothelial dysfunction, oxidative stress, and inflammation (20). Our quartile-based analyses revealed that participants in the highest quartile (Q4) of TyG-related indices had significantly higher MI risks compared to those in the lowest quartile (Q1), with HRs ranging from 1.50 to 1.90 in the fully adjusted Model 2. The incorporation of anthropometric measures in TyG-BMI, TyG-WC, and TyG-WHTR further enhanced the predictive value, as these indices capture the synergistic effects of insulin resistance and obesity-related risk factors (6). These findings are consistent with prior research demonstrating that TyG-related indices outperform traditional lipid markers in predicting cardiovascular outcomes, likely due to their ability to reflect underlying metabolic dysfunction more comprehensively (5, 21). Beyond their epidemiological association with MI, elevated TyG-related indices may reflect a constellation of metabolic disturbances that contribute to atherosclerosis and plaque instability through several plausible biological mechanisms. First, the TyG index—by incorporating both triglyceride and glucose levels—serves as a proxy for insulin resistance, a core driver of chronic low-grade inflammation. Insulin resistance leads to increased secretion of pro-inflammatory cytokines (e.g., IL-6, TNF-*α*), endothelial activation, and macrophage infiltration, all of which promote atherogenesis and plaque vulnerability (6, 22). Second, hyperglycemia and hypertriglyceridemia individually contribute to oxidative stress and endothelial dysfunction by impairing nitric oxide production, increasing advanced glycation end-products, and damaging vascular integrity. These pathophysiological changes reduce vasodilatory capacity and increase vascular stiffness, facilitating the development and progression of coronary artery disease (22, 23). Furthermore, elevated triglycerides are associated with the generation of small dense LDL particles, which have greater atherogenic potential due to enhanced arterial wall penetration and susceptibility to oxidation (22, 24). While our study did not include direct biomarker measurements of inflammation or vascular function, these mechanisms are biologically plausible pathways through which TyG-related metabolic dysregulation could lead to increased MI risk. These insights support the utility of TyG-based indices not only as risk markers but also as potential indicators of underlying pathophysiological processes amenable to lifestyle or pharmacological intervention.

Importantly, the value of TyG-related indices may lie in their practicality and accessibility as metabolic markers for population-level risk monitoring. Given that fasting glucose and triglycerides are routinely measured in clinical practice and public health screenings, TyG-based indices offer a feasible means of identifying individuals at elevated cardiometabolic risk (25). These indices may also serve as modifiable targets for lifestyle interventions, particularly nutrition-focused strategies. For instance, it is well established that diets high in refined carbohydrates, saturated fats, and added sugars contribute to hypertriglyceridemia and hyperglycemia, which would elevate the TyG index (26–28). In contrast, dietary patterns emphasizing whole grains, fiber, polyunsaturated fats, and plant-based foods are associated with improved insulin sensitivity and reduced TyG values (29, 30). While our study did not directly evaluate dietary intake, the observed associations provide epidemiological support for future intervention studies testing whether nutritional modifications that lower TyG-related indices can reduce MI risk.

Our stratified analyses by renal function status revealed a striking disparity in the associations between TyG-related indices and MI risk. In participants with preserved renal function (eGFR ≥60 mL/min/1.73 m^2^), all TyG-related indices were significantly associated with MI risk, with HRs ranging from 1.41 to 1.94 for Q4 versus Q1. However, in those with reduced renal function (eGFR <60 mL/min/1.73 m^2^), these associations were non-significant. This finding is intriguing and may be explained by several factors. First, advanced renal dysfunction is a potent independent risk factor for MI, as it induces systemic changes such as uremic toxicity, vascular calcification, and heightened inflammation, which may overshadow the contribution of insulin resistance to MI risk (11). In this context, the TyG-related indices may lose their discriminatory power, as the overwhelming burden of renal disease dominates the risk profile. Second, participants with eGFR <60 mL/min/1.73 m^2^ may have altered metabolic profiles, including changes in lipid and glucose metabolism, that disrupt the typical relationship between TyG indices and cardiovascular outcomes. For example, chronic kidney disease is often associated with lower triglyceride levels due to malnutrition or dialysis, which could weaken the TyG index’s predictive ability (31). These findings suggest that while TyG-related indices are valuable for MI risk stratification in individuals with preserved renal function, alternative markers may be needed for those with advanced renal impairment.

The L-shaped relationship between eGFR and MI risk, as observed in our restricted cubic spline analysis, further supports the notion that renal function plays a critical role in cardiovascular risk (32). The sharp increase in MI risk as eGFR falls highlights the need for early intervention in individuals with even mild renal impairment, particularly in the context of metabolic risk factors like insulin resistance.

Mediation analyses provided further insight, showing that eGFR mediated a small but significant proportion of the association between TyG-related indices and MI (e.g., 5.33% for TyG-BMI, 3.79% for TyG-WC). Conversely, TyG-related indices mediated a portion of eGFR’s effect on MI risk, with TyG-BMI mediating the largest proportion (9.78%). These findings suggest that renal function and insulin resistance are interconnected in their pathways to MI, with eGFR potentially influencing MI risk through its impact on metabolic homeostasis, and TyG indices partially explaining the cardiovascular consequences of renal dysfunction.

Our stratified analyses by sleep duration revealed a notable disparity in the associations between TyG-related indices and MI risk. Among participants with short-to-normal sleep duration, all TyG-related indices were significantly associated with MI risk. In contrast, among those with prolonged sleep, the associations were attenuated and, in some cases, non-significant. This pattern is noteworthy and may be explained by several factors.

First, prolonged sleep has been linked to a constellation of adverse health conditions, including depression, frailty, cardiovascular autonomic dysfunction, systemic inflammation, and certain chronic diseases such as heart failure and stroke (33, 34). These conditions may independently elevate MI risk, thereby overshadowing the contribution of insulin resistance as captured by TyG-related indices. Second, long sleep duration may be a marker of underlying subclinical disease processes or reduced physiological reserve, which can alter metabolic regulation and weaken the link between TyG indices and cardiovascular outcomes (35). Additionally, prolonged sleep is often associated with poorer sleep quality, circadian rhythm disruption, and reduced physical activity, all of which can contribute to cardiometabolic risk via mechanisms that are not fully captured by TyG-based measures (36, 37).

The U-shaped relationship between sleep duration and MI risk observed in our restricted cubic spline analysis further supports the complexity of this behavioral factor. Both short and long sleep durations were associated with elevated MI risk, with the lowest risk observed at intermediate durations. This suggests that deviations from optimal sleep—whether shorter or longer—may reflect underlying physiological stress or pathology, with different implications for metabolic and cardiovascular health.

Given the U-shaped association between sleep duration and MI in the full sample, mediation analyses were stratified to approximate linear relationships, and the results should be interpreted as exploratory. In participants with ≤7.5 h, sleep duration mediated a small but statistically significant proportion (approximately 2–5%) of the association between TyG-related indices and MI. Similarly, in those with >7.5 h, the mediation proportion was of comparable magnitude (2–5%), although the direct associations between TyG indices and MI were weaker. Conversely, TyG-related indices mediated 4–13% of the association between sleep duration and MI across both strata, with the highest proportion observed for TyG-WHTR in the long sleep group (12.82%). These findings suggest that sleep duration and insulin resistance may operate through partially overlapping biological pathways—potentially involving alterations in sympathetic–parasympathetic balance, systemic inflammation, endothelial function, and metabolic homeostasis—that together shape cardiovascular risk (38).

In our exploratory analyses stratified by sex and ethnicity, the associations between TyG-related indices and MI risk were generally consistent across sexes, with slightly higher effect estimates in females for the TyG index itself, but without statistically significant sex–TyG interactions. Notably, the strength of association appeared greater in Black participants compared with White participants, particularly for TyG-WHTR (HR for Q4 vs. Q1: 2.78 vs. 1.90), aligning with previous studies reporting stronger TyG–MI associations in Black populations (39). However, these findings should be interpreted cautiously due to the small number of MI events in non-White groups and the predominantly White composition of the UK Biobank. Further research in more ethnically diverse cohorts is warranted to confirm these patterns and elucidate potential biological or socio-environmental explanations.

From a preventive health perspective, our findings suggest that TyG-related indices may help identify individuals who could benefit from early lifestyle intervention, particularly those with preserved renal function and typical sleep durations. While not diagnostic tools, these indices can support public health surveillance and individualized risk assessment. Interventions targeting modifiable lifestyle factors may have the potential to lower TyG-related indices and thereby reduce cardiovascular risk. Notably, such strategies may need to be tailored in populations with comorbidities, such as CKD or sleep disorders, given their unique metabolic profiles and response patterns.

From a research perspective, our study raises several questions for future exploration. The lack of association between TyG-related indices and MI risk in participants with eGFR <60 mL/min/1.73 m^2^ warrants further investigation into the metabolic alterations associated with advanced renal disease and their impact on cardiovascular risk prediction. Additionally, the mechanisms underlying the attenuated associations in the long sleep group remain unclear and may involve complex interactions with comorbidities or lifestyle factors not fully captured in our analysis (33). Longitudinal studies with repeated measurements of TyG indices, renal function, and sleep patterns could provide deeper insights into the temporal dynamics of these relationships.

In addition, ROC analyses provided practical cut-off values for each TyG-related index, with TyG-WC showing the highest discriminative ability. Although the overall predictive performance was modest, these thresholds may assist in risk stratification when combined with other established cardiovascular risk factors.

This study has several strengths, including its large sample size, prospective design, and comprehensive adjustment for confounders, which enhance the robustness of our findings. The use of restricted cubic spline analyses allowed us to explore nonlinear relationships, providing a more nuanced understanding of the associations. Additionally, the UK Biobank’s detailed phenotyping enabled us to stratify participants by renal function and sleep duration, revealing important subgroup differences.

However, several limitations should be acknowledged. First, the TyG index and its derived indices are surrogate markers of insulin resistance and may not fully capture the complexity of insulin resistance as measured by gold-standard methods like the hyperinsulinemic-euglycemic clamp (40). Second, sleep duration was self-reported and did not capture sleep quality, sleep-disordered breathing, or other dimensions of sleep health such as timing or variability. These unmeasured factors may confound the associations observed between sleep duration and MI. Future research incorporating objective sleep measures (e.g., actigraphy or polysomnography) and standardized sleep quality assessments is warranted to elucidate the full impact of sleep on cardiovascular risk. Third, the UK Biobank cohort is predominantly of European descent, which may limit the generalizability of our findings to other ethnic groups with different metabolic and cardiovascular risk profiles. Fourth, residual confounding by unmeasured variables cannot be entirely excluded. Although we attempted to address reverse causation by excluding early events during follow-up, the possibility remains.

## Conclusion

TyG-related indices predict MI risk, modulated by renal function and sleep duration, particularly in those with preserved renal function and short/normal sleep. Integrating TyG, eGFR, and sleep assessments into clinical practice could enhance MI prevention. Future studies should investigate mechanisms behind reduced TyG predictiveness in advanced renal dysfunction and long sleep, and explore targeted interventions.

## Data Availability

The original contributions presented in the study are included in the article/Supplementary material, further inquiries can be directed to the corresponding authors.
