# Supplementary material for: Triglyceride-glucose index, renal function, sleep duration, and myocardial infarction: a UK biobank cohort study
Source: Front Nutr. 2025 Aug 29;12:1646627. doi: 10.3389/fnut.2025.1646627 (PMC12426005; doi:10.3389/fnut.2025.1646627)

Table S1. Association of TyG Index with Myocardial Infarction Risk with Additional Adjustment for BMI.

Categories	Model 1		Model 2	
	HR (95% CI)	P value	HR (95% CI)	P value
TyG				
Continuous variable per unit	1.4(1.36 - 1.44)	<0.001	1.26(1.21 - 1.32)	<0.001
Quartile				
Q1	Reference		Reference	
Q2	1.12(1.06 - 1.19)	<0.001	1.1(1.03 - 1.18)	0.007
Q3	1.28(1.21 - 1.35)	<0.001	1.21(1.13 - 1.29)	<0.001
Q4	1.58(1.5 - 1.67)	<0.001	1.39(1.29 - 1.48)	<0.001

Table S2. The capacity of TyG index to predict MI

	AUC	Optimal cut point	Sensitivity	Specificity
TyG	0.605 (0.6-0.61)	8.7	0.628 (0.62-0.637)	0.523 (0.522-0.525)
TyG-BMI	0.601 (0.596-0.606)	225.51	0.712 (0.704-0.719)	0.44 (0.439-0.442)
TyG-WC	0.642 (0.637-0.646)	777.98	0.709 (0.701-0.716)	0.501 (0.5-0.503)
TyG-WHTR	0.631 (0.627-0.636)	4.61	0.689 (0.681-0.697)	0.504 (0.502-0.505)

TyG, triglyceride-glucose index; BMI, body mass index; WC, waist circumference; WHTR, Waist-to-Height Ratio.

Table S3. Association between TyG-related indices and the risk of MI according to sex

Categories	Female		Male	
	HR (95% CI)	P value	HR (95% CI)	P value
TyG				
Continuous variable per unit	1.46(1.34 - 1.58)	<0.001	1.26(1.2 - 1.32)	<0.001
Quartile				
Q1	Reference		Reference	
Q2	1.13(1 - 1.28)	0.051	1.09(1 - 1.19)	0.039
Q3	1.32(1.16 - 1.49)	<0.001	1.19(1.1 - 1.29)	<0.001
Q4	1.66(1.46 - 1.88)	<0.001	1.37(1.27 - 1.48)	<0.001
TyG-BMI				
Continuous variable per unit	1(1 - 1)	<0.001	1(1 - 1)	<0.001
Quartile				
Q1	Reference		Reference	
Q2	1.15(1.01 - 1.29)	0.029	1.29(1.18 - 1.41)	<0.001
Q3	1.37(1.21 - 1.55)	<0.001	1.38(1.26 - 1.5)	<0.001
Q4	1.53(1.35 - 1.73)	<0.001	1.69(1.55 - 1.84)	<0.001
TyG-WC				
Continuous variable per unit	1(1 - 1)	<0.001	1(1 - 1)	<0.001
Quartile				
Q1	Reference		Reference	
Q2	1.29(1.15 - 1.44)	<0.001	1.3(1.13 - 1.49)	<0.001
Q3	1.37(1.22 - 1.55)	<0.001	1.56(1.37 - 1.78)	<0.001

Q4	1.74(1.53 - 1.98)	<0.001	1.75(1.53 - 1.99)	<0.001
TyG-WHTR				
Continuous variable per unit	1.28(1.22 - 1.35)	<0.001	1.27(1.23 - 1.32)	<0.001
Quartile				
Q1	Reference		Reference	
Q2	1.32(1.17 - 1.5)	<0.001	1.35(1.22 - 1.5)	<0.001
Q3	1.47(1.3 - 1.67)	<0.001	1.61(1.45 - 1.78)	<0.001
Q4	1.81(1.6 - 2.05)	<0.001	1.85(1.67 - 2.05)	<0.001

TyG, triglyceride-glucose index; BMI, body mass index; WC, waist circumference; WHTR, Waist-to-Height Ratio. HR, hazard ratios; CI, confidence intervals;

Table S4. Association between TyG-related indices and the risk of MI according to Ethnicity

Categories	White		Black		Asian		Others	
	HR (95% CI)	P value	HR (95% CI)	P value	HR (95% CI)	P value	HR (95% CI)	P value
TyG								
Continuous variable per unit	1.32(1.27 - 1.38)	<0.001	1.32(1.06 - 1.65)	0.013	0.8(0.38 - 1.71)	0.572	0.81(0.62 - 1.05)	0.116
Quartile								
Q1	Reference		Reference		Reference		Reference	
Q2	1.14(1.06 - 1.22)	0.001	1.28(0.79 - 2.06)	0.316	0.12(0.02 - 0.93)	0.042	1.38(0.73 - 2.6)	0.327
Q3	1.27(1.18 - 1.36)	<0.001	1.53(0.97 - 2.4)	0.066	0.62(0.19 - 2.01)	0.425	1.66(0.9 - 3.07)	0.106
Q4	1.49(1.39 - 1.59)	<0.001	1.72(1.11 - 2.67)	0.016	0.62(0.16 - 2.34)	0.477	1.87(1.02 - 3.45)	0.044
TyG-BMI								
Continuous variable per unit	1(1 - 1)	<0.001	1(1 - 1.01)	0.003	1.01(1 - 1.01)	0.14	1 - 1.01(0.393)	0.116
Quartile								
Q1	Reference		Reference		Reference		Reference	
Q2	1.28(1.19 -	<0.001	1.37(0.91 -	0.135	0(0 - Inf)	0.996	0.98(0.53 -	0.954

	1.38)		2.09)				1.83)	
Q3	1.4(1.3 - 1.5)	<0.001	1.78(1.19 - 2.66)	0.005	1.41(0.38 - 5.27)	0.608	1.13(0.62 - 2.06)	0.688
Q4	1.69(1.57 - 1.82)	<0.001	1.74(1.13 - 2.67)	0.012	1.21(0.31 - 4.75)	0.786	1.55(0.86 - 2.8)	0.145
TyG-WC								
Continuous variable per unit	1(1 - 1)	<0.001	1(1 - 1)	0.002	1(1 - 1.01)	0.129	1(1 - 1)	0.127
Quartile								
Q1	Reference		Reference		Reference		Reference	
Q2	1.37(1.26 - 1.49)	<0.001	1.63(0.92 - 2.9)	0.097	0.3(0.06 - 1.37)	0.119	1.17(0.58 - 2.38)	0.654
Q3	1.6(1.47 - 1.74)	<0.001	2.07(1.18 - 3.66)	0.012	0.56(0.15 - 2.09)	0.391	1.42(0.71 - 2.85)	0.317
Q4	1.81(1.66 - 1.97)	<0.001	2.36(1.32 - 4.24)	0.004	1.08(0.31 - 3.75)	0.908	1.51(0.73 - 3.1)	0.262
TyG-WHTR								
Continuous variable per unit	1.29(1.25 - 1.32)	<0.001	1.35(1.14 - 1.61)	0.001	1.42(0.82 - 2.46)	0.213	1.26(0.98 - 1.63)	0.076
Quartile								

Q1	Reference		Reference		Reference		Reference	
Q2	1.41(1.3 - 1.53)	<0.001	1.86(0.97 - 3.53)	0.06	0.13(0.01 - 1.2)	0.072	0.72(0.36 - 1.43)	0.352
Q3	1.64(1.51 - 1.77)	<0.001	2.26(1.21 - 4.23)	0.01	0.83(0.24 - 2.9)	0.773	1.2(0.66 - 2.21)	0.552
Q4	1.9(1.76 - 2.06)	<0.001	2.78(1.49 - 5.21)	0.001	1.06(0.31 - 3.67)	0.927	1.24(0.66 - 2.3)	0.506

TyG, triglyceride-glucose index; BMI, body mass index; WC, waist circumference; WHTR, Waist-to-Height Ratio. HR, hazard ratios; CI, confidence intervals;

Table S5. Association between TyG-related indices and the risk of MI according to kidney function after excluding participants who developed MI within 2 years of baseline.

Categories	Total		eGFR⩾90		eGFR 60-90		eGFR<60	
	HR (95% CI)	P value	HR (95% CI)	P value	HR (95% CI)	P value	HR (95% CI)	P value
TyG								
Continuous variable per unit	1.33(1.27 - 1.38)	<0.001	1.36(1.29 - 1.43)	<0.001	1.26(1.17 - 1.35)	<0.001	1.01(0.8 - 1.27)	0.92
Quartile								
Q1	Reference		Reference		Reference		Reference	
Q2	1.14(1.06 - 1.22)	<0.001	1.13(1.04 - 1.24)	0.006	1.14(1.01 - 1.28)	0.037	0.89(0.59 - 1.35)	0.589
Q3	1.27(1.19 - 1.36)	<0.001	1.3(1.2 - 1.42)	<0.001	1.24(1.1 - 1.39)	<0.001	0.63(0.42 - 0.95)	0.027
Q4	1.49(1.39 - 1.59)	<0.001	1.53(1.41 - 1.66)	<0.001	1.42(1.26 - 1.59)	<0.001	0.85(0.58 - 1.24)	0.401
TyG-BMI								
Continuous variable per unit	1(1 - 1)	<0.001	1(1 - 1)	<0.001	1(1 - 1)	<0.001	1(1 - 1)	0.961
Quartile								
Q1	Reference		Reference		Reference		Reference	

Q2	1.26(1.17 - 1.35)	<0.001	1.24(1.14 - 1.35)	<0.001	1.23(1.08 - 1.4)	0.001	1.16(0.71 - 1.89)	0.548
Q3	1.36(1.27 - 1.46)	<0.001	1.37(1.26 - 1.49)	<0.001	1.29(1.13 - 1.46)	<0.001	1.16(0.73 - 1.85)	0.52
Q4	1.59(1.48 - 1.71)	<0.001	1.6(1.47 - 1.75)	<0.001	1.5(1.32 - 1.7)	<0.001	1.2(0.76 - 1.88)	0.432
TyG-WC								
Continuous variable per unit	1(1 - 1)	<0.001	1(1 - 1)	0	1(1 - 1)	<0.001	1(1 - 1)	0.813
Quartile								
Q1	Reference		Reference		Reference		Reference	
Q2	1.35(1.24 - 1.47)	<0.001	1.35(1.22 - 1.49)	<0.001	1.34(1.16 - 1.56)	<0.001	0.88(0.52 - 1.48)	0.62
Q3	1.55(1.43 - 1.69)	<0.001	1.59(1.44 - 1.76)	<0.001	1.47(1.26 - 1.7)	<0.001	0.91(0.55 - 1.5)	0.718
Q4	1.73(1.59 - 1.88)	<0.001	1.74(1.57 - 1.93)	<0.001	1.66(1.43 - 1.93)	<0.001	0.99(0.6 - 1.62)	0.954
TyG-WHTR								
Continuous variable per unit	1.26(1.22 - 1.29)	<0.001	1.26(1.22 - 1.31)	<0.001	1.24(1.18 - 1.31)	<0.001	1.04(0.9 - 1.21)	0.577

Quartile								
Q1	Reference		Reference		Reference		Reference	
Q2	1.38(1.27 - 1.49)	<0.001	1.39(1.26 - 1.53)	<0.001	1.32(1.15 - 1.52)	<0.001	1.14(0.65 - 1.97)	0.65
Q3	1.59(1.47 - 1.72)	<0.001	1.61(1.46 - 1.77)	<0.001	1.49(1.3 - 1.71)	<0.001	1.31(0.78 - 2.21)	0.307
Q4	1.81(1.67 - 1.95)	<0.001	1.86(1.69 - 2.04)	<0.001	1.66(1.45 - 1.91)	<0.001	1.29(0.77 - 2.15)	0.332

TyG, triglyceride-glucose index; BMI, body mass index; WC, waist circumference; WHTR, Waist-to-Height Ratio. HR, hazard ratios; CI, confidence intervals;

Table S6. Association between TyG-related indices and the risk of MI according to sleep durattion after excluding participants who developed MI within 2 years of baseline.

Categories	Total		Short		Normal		Long	
	HR (95% CI)	P value	HR (95% CI)	P value	HR (95% CI)	P value	HR (95% CI)	P value
TyG								
Continuous variable per unit	1.18(1.13 - 1.22)	<0.001	1.4(1.29 - 1.53)	<0.001	1.46(1.38 - 1.54)	<0.001	1.26(1 - 1.58)	0.051
Quartile								
Q1	Reference		Reference		Reference		Reference	
Q2	1.12(1.04 - 1.2)	0.002	1.14(0.99 - 1.3)	0.06	1.13(1.04 - 1.23)	0.005	0.67(0.41 - 1.1)	0.113
Q3	1.19(1.11 - 1.27)	<0.001	1.11(0.97 - 1.26)	0.126	1.22(1.12 - 1.32)	<0.001	1.07(0.7 - 1.65)	0.751
Q4	1.3(1.21 - 1.39)	<0.001	1.26(1.11 - 1.43)	<0.001	1.32(1.22 - 1.43)	<0.001	1.12(0.74 - 1.7)	0.582
TyG-BMI								
Continuous variable per unit	1(1 - 1)	<0.001	1(1 - 1)	<0.001	1(1 - 1)	<0.001	1(1 - 1)	0.751
Quartile								

Q1	Reference		Reference		Reference		Reference	
Q2	1.29(1.2 - 1.38)	<0.001	1.47(1.27 - 1.69)	<0.001	1.24(1.14 - 1.34)	<0.001	1.26(0.77 - 2.05)	0.358
Q3	1.36(1.26 - 1.45)	<0.001	1.47(1.28 - 1.69)	<0.001	1.32(1.22 - 1.44)	<0.001	1.31(0.82 - 2.08)	0.26
Q4	1.33(1.24 - 1.43)	<0.001	1.32(1.15 - 1.52)	<0.001	1.34(1.24 - 1.46)	<0.001	1.23(0.8 - 1.91)	0.345
TyG-WC								
Continuous variable per unit	1(1 - 1)	0.014	1(1 - 1)	<0.001	1(1 - 1)	<0.001	1(1 - 1)	0.689
Quartile								
Q1	Reference		Reference		Reference		Reference	
Q2	1.23(1.13 - 1.33)	<0.001	1.25(1.07 - 1.47)	0.006	1.21(1.1 - 1.34)	<0.001	1.35(0.71 - 2.57)	0.354
Q3	1.37(1.26 - 1.49)	<0.001	1.39(1.18 - 1.62)	<0.001	1.36(1.24 - 1.5)	<0.001	1.62(0.88 - 2.99)	0.122
Q4	1.3(1.2 - 1.41)	<0.001	1.26(1.07 - 1.47)	0.005	1.32(1.2 - 1.46)	<0.001	1.26(0.69 - 2.3)	0.45
TyG-WHTR								
Continuous variable per	1.06(1.03 - 1.09)	<0.001	1.03(1 - 1.07)	0.013	1.08(1.04 - 1.12)	<0.001	1(0.87 - 1.16)	0.962

unit								
Quartile								
Q1	Reference		Reference		Reference		Reference	
Q2	1.3(1.2 - 1.4)	<0.001	1.45(1.24 - 1.69)	<0.001	1.25(1.14 - 1.37)	<0.001	1.45(0.78 - 2.7)	0.244
Q3	1.38(1.28 - 1.49)	<0.001	1.51(1.3 - 1.77)	<0.001	1.33(1.22 - 1.46)	<0.001	1.48(0.81 - 2.69)	0.199
Q4	1.35(1.25 - 1.46)	<0.001	1.39(1.19 - 1.62)	<0.001	1.34(1.22 - 1.46)	<0.001	1.45(0.81 - 2.59)	0.206

TyG, triglyceride-glucose index; BMI, body mass index; WC, waist circumference; WHTR, Waist-to-Height Ratio. HR, hazard ratios; CI, confidence intervals;

Table S7. Association between TyG-related indices and the risk of MI according to kidney function with further adjustment for glucose-lowering drugs and lipid-lowering medications.

Categories	Total		eGFR≥90		eGFR 60-90		eGFR<60	
	HR (95% CI)	P value	HR (95% CI)	P value	HR (95% CI)	P value	HR (95% CI)	P value
TyG								
Continuous variable per unit	1.33(1.27 - 1.38)	<0.001	1.36(1.29 - 1.43)	<0.001	1.26(1.17 - 1.35)	<0.001	1.01(0.8 - 1.27)	0.92
Quartile								
Q1	Reference		Reference		Reference		Reference	
Q2	1.14(1.06 - 1.22)	<0.001	1.13(1.04 - 1.24)	0.006	1.14(1.01 - 1.28)	0.037	0.89(0.59 - 1.35)	0.589
Q3	1.27(1.19 - 1.36)	<0.001	1.3(1.2 - 1.42)	<0.001	1.24(1.1 - 1.39)	<0.001	0.63(0.42 - 0.95)	0.027
Q4	1.49(1.39 - 1.59)	<0.001	1.53(1.41 - 1.66)	<0.001	1.42(1.26 - 1.59)	<0.001	0.85(0.58 - 1.24)	0.401
TyG-BMI								
Continuous variable per unit	1(1 - 1)	<0.001	1(1 - 1)	<0.001	1(1 - 1)	<0.001	1(1 - 1)	0.961
Quartile								
Q1	Reference		Reference		Reference		Reference	

Q2	1.26(1.17 - 1.35)	<0.001	1.24(1.14 - 1.35)	<0.001	1.23(1.08 - 1.4)	0.001	1.16(0.71 - 1.89)	0.548
Q3	1.36(1.27 - 1.46)	<0.001	1.37(1.26 - 1.49)	<0.001	1.29(1.13 - 1.46)	<0.001	1.16(0.73 - 1.85)	0.52
Q4	1.59(1.48 - 1.71)	<0.001	1.6(1.47 - 1.75)	<0.001	1.5(1.32 - 1.7)	<0.001	1.2(0.76 - 1.88)	0.432
TyG-WC								
Continuous variable per unit	1(1 - 1)	<0.001	1(1 - 1)	<0.001	1(1 - 1)	<0.001	1(1 - 1)	0.813
Quartile								
Q1	Reference		Reference		Reference		Reference	
Q2	1.35(1.24 - 1.47)	<0.001	1.35(1.22 - 1.49)	<0.001	1.34(1.16 - 1.56)	<0.001	0.88(0.52 - 1.48)	0.62
Q3	1.55(1.43 - 1.69)	<0.001	1.59(1.44 - 1.76)	<0.001	1.47(1.26 - 1.7)	<0.001	0.91(0.55 - 1.5)	0.718
Q4	1.73(1.59 - 1.88)	<0.001	1.74(1.57 - 1.93)	<0.001	1.66(1.43 - 1.93)	<0.001	0.99(0.6 - 1.62)	0.954
TyG-WHTR								
Continuous variable per unit	1.26(1.22 - 1.29)	<0.001	1.26(1.22 - 1.31)	<0.001	1.24(1.18 - 1.31)	<0.001	1.04(0.9 - 1.21)	0.577

Quartile								
Q1	Reference		Reference		Reference		Reference	
Q2	1.38(1.27 - 1.49)	<0.001	1.39(1.26 - 1.53)	<0.001	1.32(1.15 - 1.52)	<0.001	1.14(0.65 - 1.97)	0.65
Q3	1.59(1.47 - 1.72)	<0.001	1.61(1.46 - 1.77)	<0.001	1.49(1.3 - 1.71)	<0.001	1.31(0.78 - 2.21)	0.307
Q4	1.81(1.67 - 1.95)	<0.001	1.86(1.69 - 2.04)	<0.001	1.66(1.45 - 1.91)	<0.001	1.29(0.77 - 2.15)	0.332

TyG, triglyceride-glucose index; BMI, body mass index; WC, waist circumference; WHTR, Waist-to-Height Ratio. HR, hazard ratios; CI, confidence intervals;

Table S8. Association between TyG-related indices and the risk of MI according to sleep duration with further adjustment for glucose-lowering drugs and lipid-lowering medications.

Categories	Total		Short		Normal		Long	
	HR (95% CI)	P value	HR (95% CI)	P value	HR (95% CI)	P value	HR (95% CI)	P value
TyG								
Continuous variable per unit	1.33(1.27 - 1.38)	<0.001	1.26(1.17 - 1.36)	<0.001	1.34(1.27 - 1.4)	<0.001	1.23(0.97 - 1.55)	0.085
Quartile								
Q1	Reference		Reference		Reference		Reference	
Q2	1.14(1.06 - 1.22)	<0.001	1.1(0.97 - 1.26)	0.147	1.16(1.07 - 1.26)	<0.001	0.68(0.41 - 1.11)	0.121
Q3	1.27(1.19 - 1.36)	<0.001	1.15(1.01 - 1.3)	0.042	1.32(1.22 - 1.43)	<0.001	1(0.65 - 1.53)	0.986
Q4	1.49(1.39 - 1.59)	<0.001	1.4(1.24 - 1.59)	<0.001	1.51(1.4 - 1.64)	<0.001	1.05(0.69 - 1.6)	0.804
TyG-BMI								
Continuous variable per unit	1(1 - 1)	<0.001	1(1 - 1)	<0.001	1(1 - 1)	<0.001	1(1 - 1)	0.54
Quartile								
Q1	Reference		Reference		Reference		Reference	

Q2	1.26(1.17 - 1.35)	<0.001	1.36(1.18 - 1.57)	<0.001	1.23(1.13 - 1.34)	<0.001	0.99(0.61 - 1.61)	0.958
Q3	1.36(1.27 - 1.46)	<0.001	1.4(1.22 - 1.61)	<0.001	1.36(1.25 - 1.47)	<0.001	0.92(0.58 - 1.46)	0.723
Q4	1.59(1.48 - 1.71)	<0.001	1.51(1.31 - 1.73)	<0.001	1.61(1.48 - 1.75)	<0.001	1.18(0.76 - 1.84)	0.469
TyG-WC								
Continuous variable per unit	1(1 - 1)	<0.001	1(1 - 1)	<0.001	1(1 - 1)	<0.001	1(1 - 1)	0.568
Quartile								
Q1	Reference		Reference		Reference		Reference	
Q2	1.35(1.24 - 1.47)	<0.001	1.27(1.08 - 1.49)	0.004	1.37(1.24 - 1.51)	<0.001	1.56(0.82 - 2.96)	0.171
Q3	1.55(1.43 - 1.69)	<0.001	1.48(1.26 - 1.73)	<0.001	1.57(1.42 - 1.73)	<0.001	1.61(0.87 - 2.99)	0.131
Q4	1.73(1.59 - 1.88)	<0.001	1.58(1.34 - 1.85)	<0.001	1.75(1.59 - 1.94)	<0.001	1.62(0.88 - 3)	0.123
TyG-WHTR								
Continuous variable per unit	1.26(1.22 - 1.29)	<0.001	1.19(1.13 - 1.25)	<0.001	1.27(1.23 - 1.32)	<0.001	1.09(0.93 - 1.27)	0.281

Quartile								
Q1	Reference		Reference		Reference		Reference	
Q2	1.38(1.27 - 1.49)	<0.001	1.52(1.29 - 1.78)	<0.001	1.32(1.21 - 1.45)	<0.001	1.66(0.89 - 3.08)	0.109
Q3	1.59(1.47 - 1.72)	<0.001	1.67(1.43 - 1.95)	<0.001	1.55(1.42 - 1.7)	<0.001	1.36(0.74 - 2.47)	0.319
Q4	1.81(1.67 - 1.95)	<0.001	1.78(1.53 - 2.08)	<0.001	1.78(1.62 - 1.95)	<0.001	1.77(0.98 - 3.17)	0.057

TyG, triglyceride-glucose index; BMI, body mass index; WC, waist circumference; WHTR, Waist-to-Height Ratio. HR, hazard ratios; CI, confidence intervals;

Table S9. The mediating effect of renal function and sleep duration on the association of insulin resistance with MI.

Mediators	Direct effect		Indirect effect		Proportion mediated (%)
TyG	Coefficients (95%CI)	P	Coefficients (95%CI)	P	
eGFR	-2.192 (-2.521 to -1.840)	<0.001	-0.046 (-0.057 to -0.030)	<0.001	2.05% (1.43% to 3.00%)
Sleep duration					
≤7.5h	-2.192 (-2.596 to -1.840)	<0.001	-0.047 (-0.060 to -0.040)	<0.001	2.12% (1.69% to 3.00%)
>7.5h	-2.258 (-2.505 to -1.950)	<0.001	-0.088 (-0.108 to -0.060)	<0.001	3.75% (2.61% to 5.00%)
TyG-BMI					
eGFR	-0.027 (-0.029 to -0.020)	<0.001	-0.00150 (-0.00155 to 0.00)	<0.001	5.33% (4.57% to 6.00%)
Sleep duration					
≤7.5h	-0.026 (-0.030 to -0.020)	<0.001	-0.001 (-0.002 to 0.000)	<0.001	4.72% (3.81% to 6.00%)
>7.5h	-0.031 (-0.034 to -0.030)	<0.001	-0.001 (-0.002 to 0.000)	<0.001	4.25% (3.28% to 6.00%)
TyG-WC					
eGFR	-0.010 (-0.011 to -0.010)	<0.001	-3.91 e-04 (-4.54 e-04 to 0.00)	<0.001	3.79% (3.19% to 5.00%)
Sleep duration					
≤7.5h	0.010 (-0.011 to -0.010)	<0.001	-0.000417 (-0.000492 to 0.00)	<0.001	4.05% (3.29% to 5.00%)
>7.5h	-0.011 (-0.012 to -0.010)	<0.001	-0.000541(-0.000647 to 0.00)	<0.001	4.79% (3.71% to 6.00%)

TyG-WHTR					
eGFR	-1.991 (-2.246 to -1.870)	<0.001	-0.0496 (-0.0545 to -0.04)	<0.001	2.43% (1.96% to 3.00%)
Sleep duration					
≤7.5h	-1.934 (-2.079 to -1.700)	<0.001	-0.078 (-0.086 to 0.000)	<0.001	3.89% (3.15% to 4.00%)
>7.5h	-2.159 (-2.392 to -1.840)	<0.001	-0.093 (-0.110 to -0.070)	<0.001	4.13% (2.99% to 5.00%)

TyG, triglyceride-glucose index; BMI, body mass index; WC, waist circumference; WHTR, Waist-to-Height Ratio.

Table S10. The mediating effect of insulin resistance indices on the associations of renal function and sleep duration with MI

Mediators	Direct effect		Indirect effect		Proportion mediated (%)
eGFR	Coefficients (95%CI)	P	Coefficients (95%CI)	P	
TyG	0.079 (0.073 to 0.09)	<0.001	0.002 (0.002 to 0.00)	<0.001	3.11% (2.81% to 4.00%)
TyG-BMI	0.074 (0.069 to 0.08)	<0.001	0.008 (0.007 to 0.01)	<0.001	9.78% (8.13% to 12.00%)
TyG-WC	0.076 (0.067 to 0.08)	<0.001	0.005 (0.005 to 0.01)	<0.001	6.85% (6.08% to 8.00%)
TyG-WHTR	0.076(0.065 to 0.09)	<0.001	0.005 (0.004 to 0.01	<0.001	6.28% (5.77% to 7.00%)
Sleep duration ≤7.5h					
TyG	1.170 (1.040 to 1.420)	<0.001	0.048 (0.036 to 0.070)	<0.001	3.97% (2.91% to 6.00%)
TyG-BMI	1.097 (0.973 to 1.350)	<0.001	0.125 (0.100 to 0.140)	<0.001	10.19% (7.74% to 12.00%)
TyG-WC	1.102 (0.889 to 1.300)	<0.001	0.111 (0.105 to 0.120)	<0.001	9.15% (7.91% to 11.00%)
TyG-WHTR	1.063 (0.993 to 1.280)	<0.001	0.152 (0.135 to 0.170)	<0.001	12.50% (11.40% to 15.00%)
Sleep duration >7.5h					
TyG	-1.413 (-1.751 to -1.090)	<0.001	-0.091 (-0.112 to -0.070)	<0.001	6.04% (4.52% to 8.00%)
TyG-BMI	-1.364 (-1.691 to -1.040)	<0.001	-0.149 (-0.174 to -0.130)	<0.001	9.83% (8.40% to 13.00%)
TyG-WC	-1.361 (-1.619 to -1.180)	<0.001	-0.151 (-0.176 to -0.130)	<0.001	9.97% (8.38% to 12.00%)
TyG-WHTR	-1.321 (-1.718 to -0.950)	<0.001	-0.194 (-0.212 to -0.130)	<0.001	12.82% (7.86% to 15.00%)

TyG, triglyceride-glucose index; BMI, body mass index; WC, waist circumference; WHTR, Waist-to-Height Ratio.

Table S11. Bonferroni correction

	Non-Myocardial infarction	Myocardial infarction	P	Short vs normal	Normal vs long	Short vs long
Sample size	402273	13484				
Sleep time, n (%)			<0.001			
Short	98657 (24.5)	3668 (27.2)		<0.001		
Normal	296674 (73.7)	9420 (69.9)			<0.001	
Long	6942 (1.7)	396 (2.9)				<0.001
				eGFR<60 vs eGFR 60-90	eGFR 60-90 vs eGFR>90	eGFR<60 vs eGFR 90
Renal function			<0.001			
eGFR<60	5463 (1.4)	458 (3.4)		<0.001		
eGFR 60-90	114457 (28.5)	4699 (34.9)			<0.001	
eGFR>90	282306 (70.2)	8325 (61.7)				<0.001

Figure S1. Flowchart illustrating the participant selection process for the study

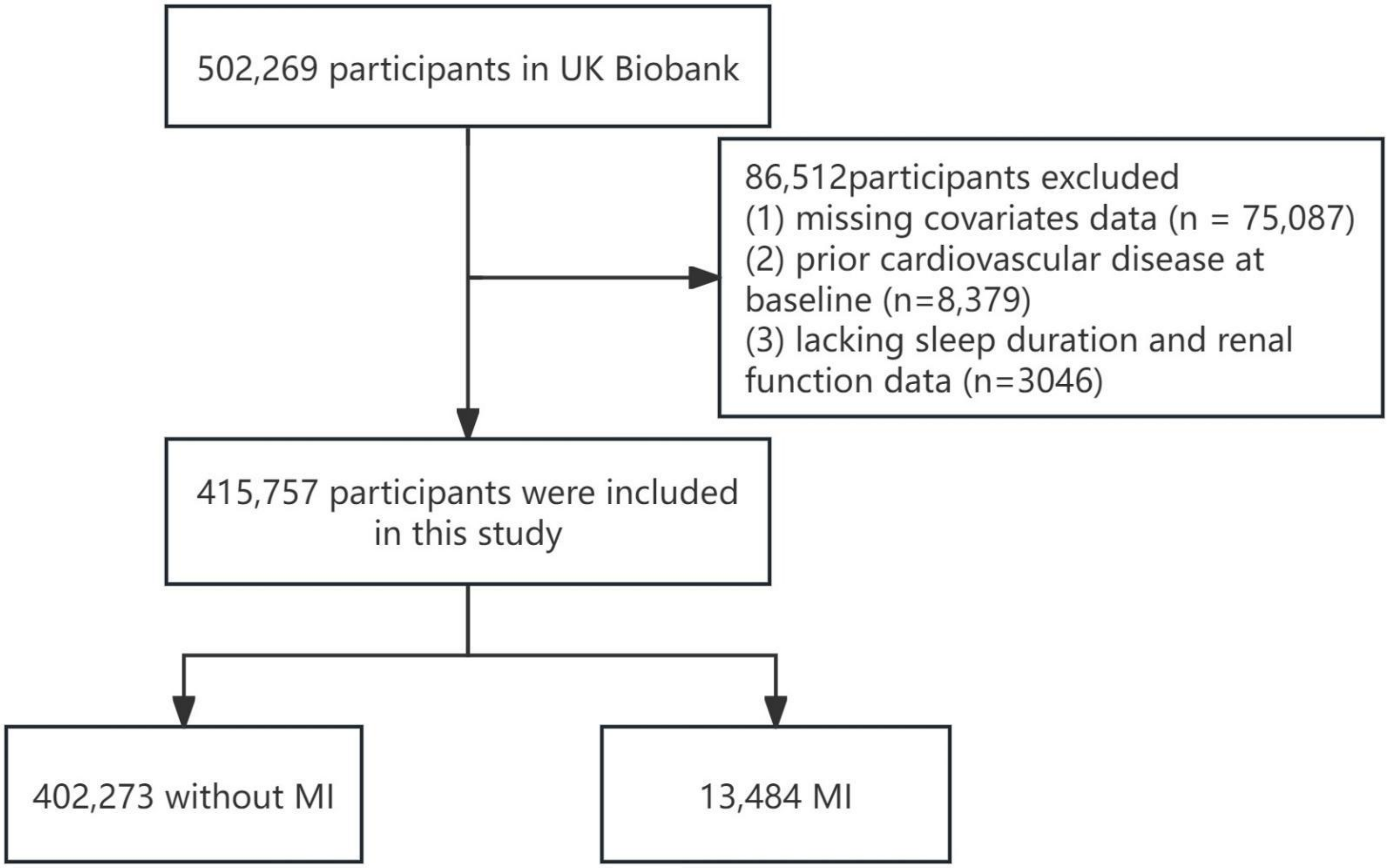

Figure S2. The Kaplan-Meier analysis for myocardial infarction was based on TyG, TyG-BMI, TyG-WC, and TyG-WHTR indexes quartiles for total participants, participants with eGFR⩾90 mL/min/1.73 m², participants with eGFR 60-90 mL/min/1.73 m², and participants with eGFR<60 mL/min/1.73 m².

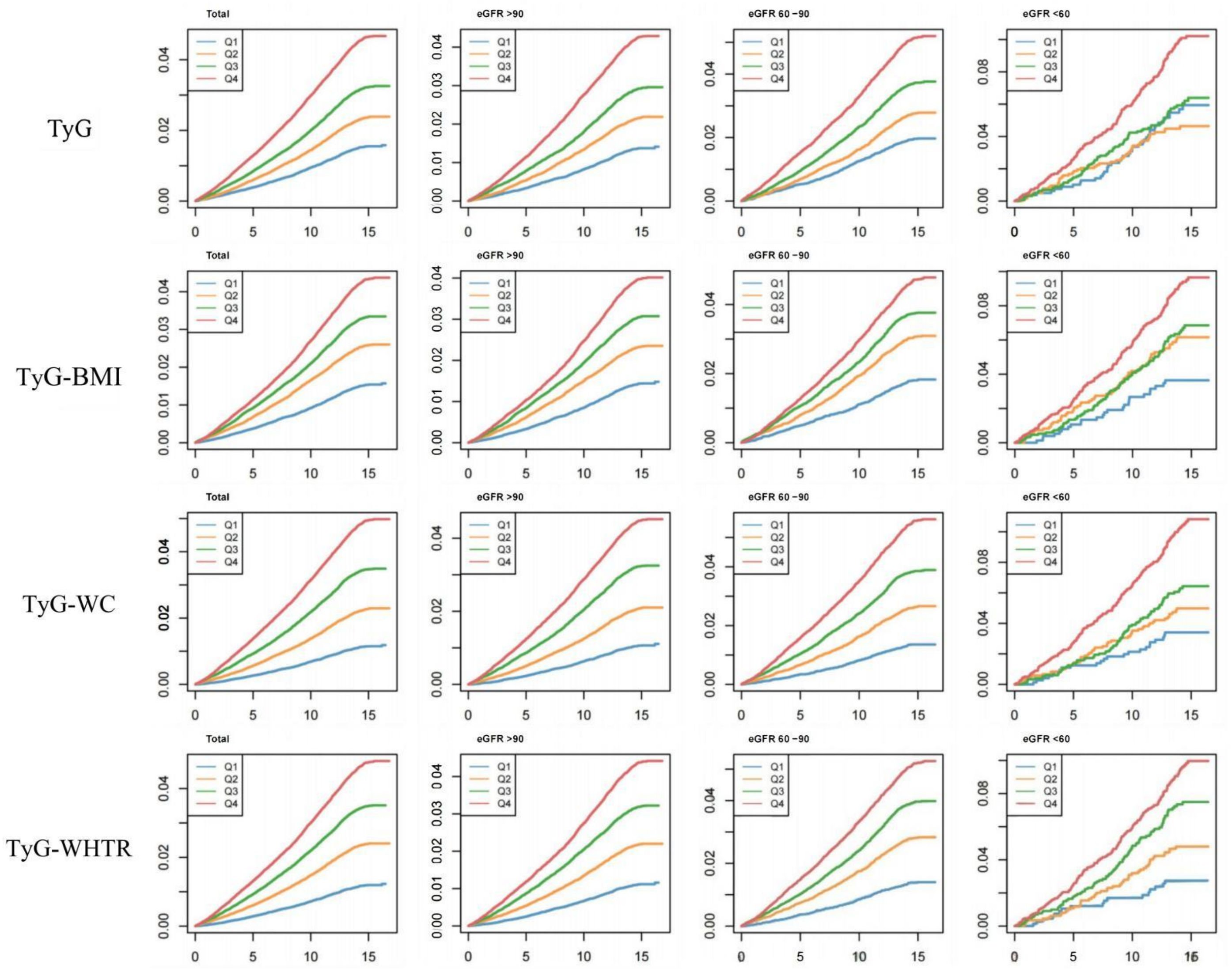

Figure S3. Restricted cubic spline analyses showing the dose-response relationships between TyG-related indices and myocardial infarction risk, stratified by renal function status.

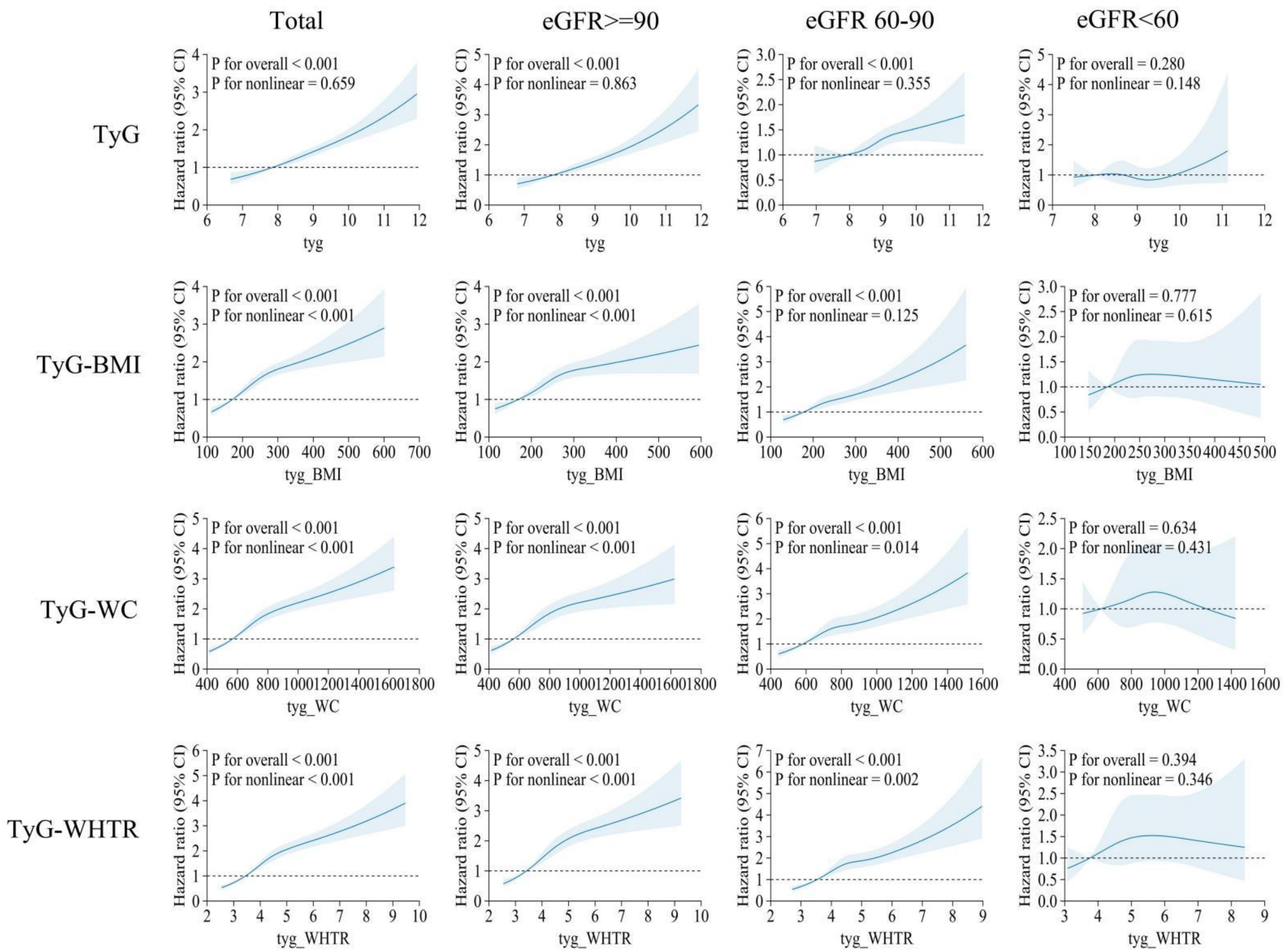

Figure S4. Kaplan-Meier survival curves showing the cumulative incidence of myocardial infarction across quartiles of TyG-related indices, stratified by sleep duration categories.

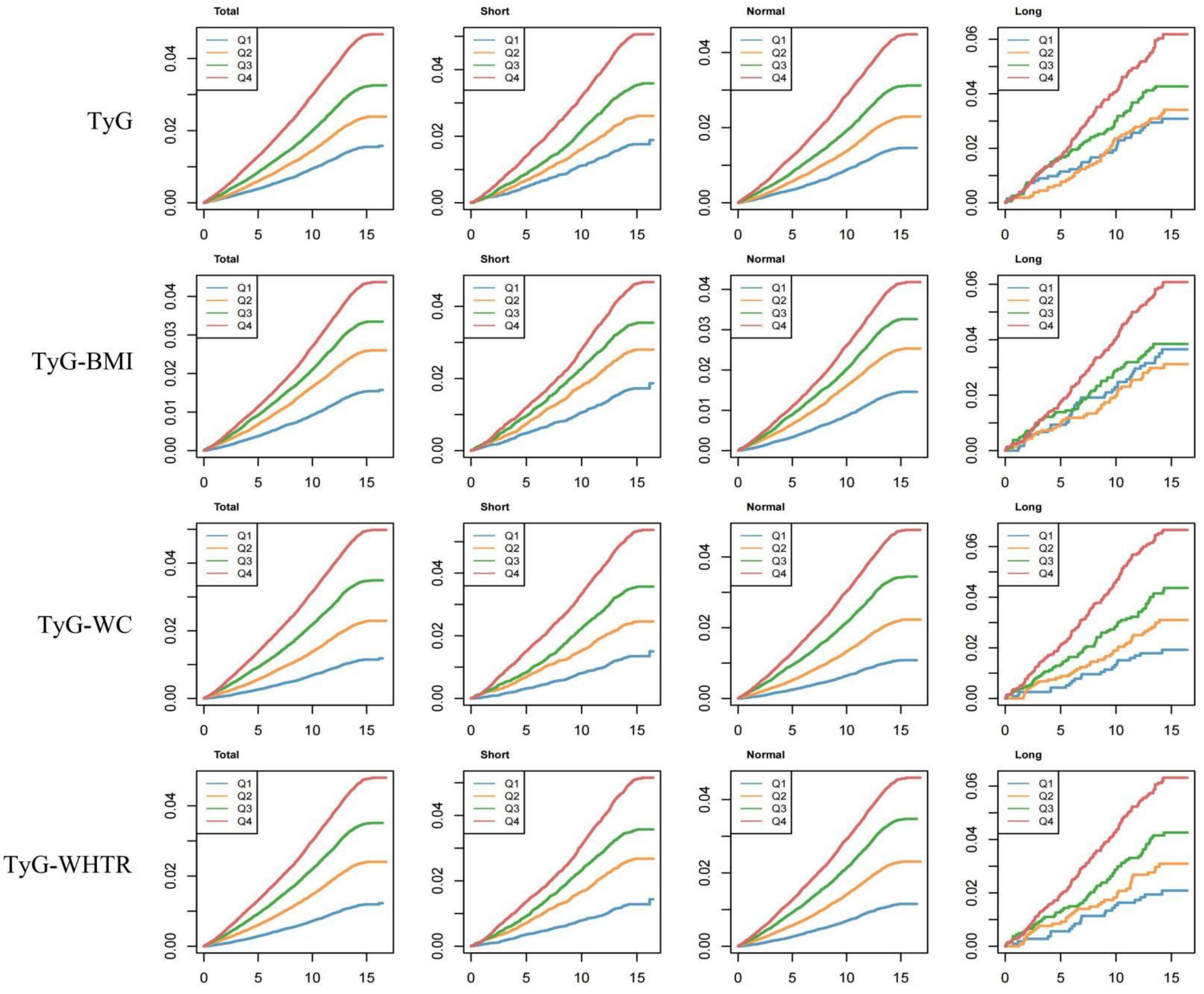

Figure S5. Restricted cubic spline analyses showing the dose-response relationships between TyG-related indices and myocardial infarction risk, stratified by sleep duration categories.

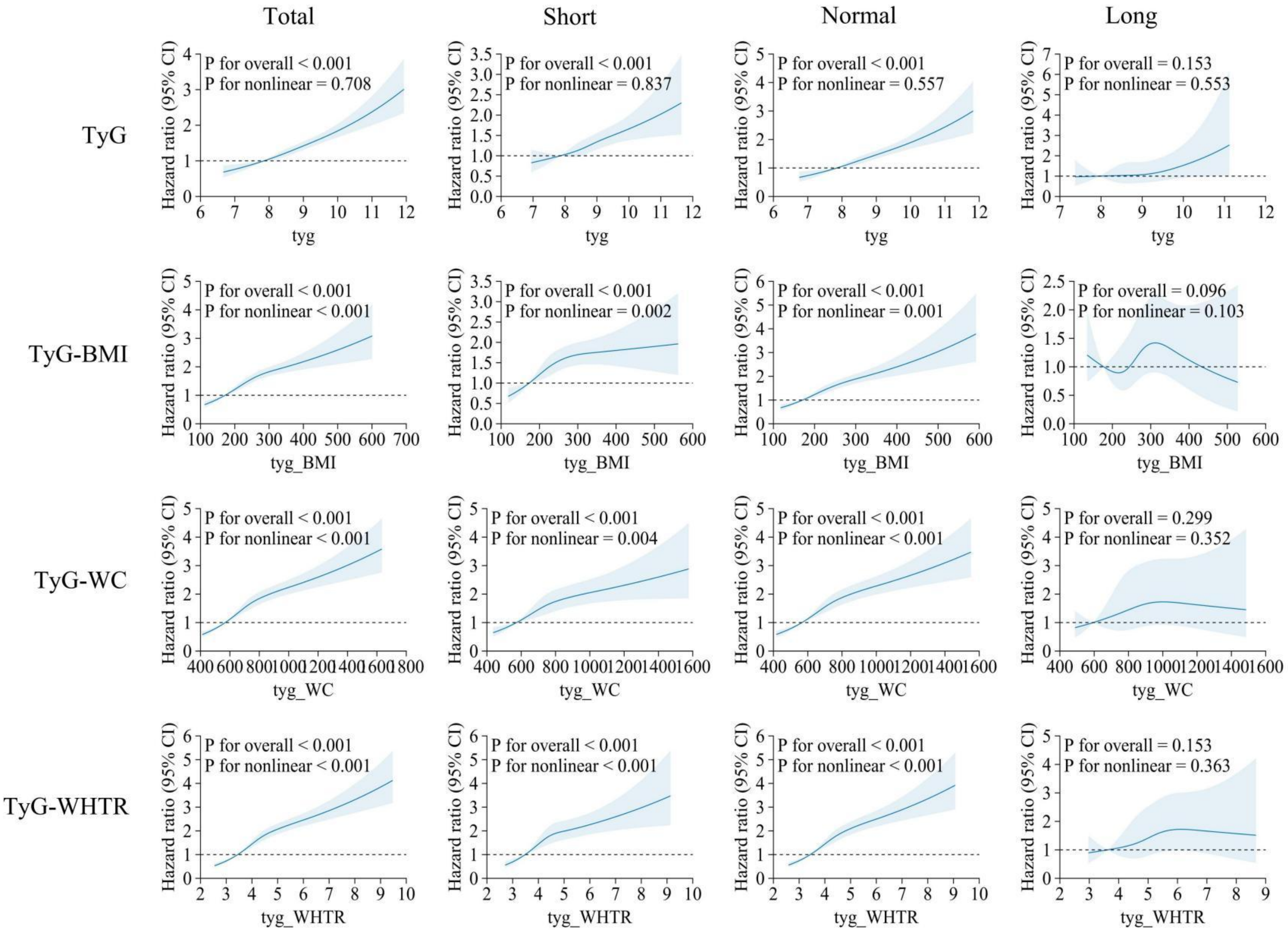

Supplement: Supplementary file 1 [file Data_Sheet_1.pdf]
